# Case Report: Pleomorphic adenoma of the breast: diagnostic pitfalls and a novel PLAG1::FTO fusion

**DOI:** 10.3389/fonc.2026.1722310

**Published:** 2026-03-06

**Authors:** Jing Lian, Mengqin Yang, Peng Bu, Junfeng Jiang, Chun Wu, Yanfeng Xi

**Affiliations:** 1 Department of Pathology, Shanxi Province Cancer Hospital/Shanxi Hospital Affiliated to Cancer Hospital, Chinese Academy of Medical Sciences/Cancer Hospital Affiliated to Shanxi Medical University, Taiyuan, China; 2 Abcarta Medtech Co. Ltd., Suzhou, China

**Keywords:** breast, pleomorphic adenoma, chromosome conformation capture, RNA sequencing, PLAG1::FTO

## Abstract

Pleomorphic adenoma (PA) of the breast and its malignant transformation represent extremely rare clinical entities, and the molecular alterations underlying this tumor type remain largely uninvestigated to date. Herein, we report a diagnostic challenge posed by a case of mammary pleomorphic adenoma, which was initially misdiagnosed as mucinous carcinoma during frozen section examination; the definitive diagnosis was ultimately confirmed based on a comprehensive analysis of the surgical resection specimen. As implied by the term “pleomorphic,” this tumor exhibits remarkable phenotypic heterogeneity. Therefore, a thorough understanding of the morphological features of rare breast PA is crucial for mitigating the risk of misdiagnosis in clinical practice. To explore the genetic characteristics of this tumor, we employed High-throughput Chromosome Conformation Capture (Hi-C) and RNA sequencing (RNA-Seq) to detect potential gene rearrangements. Importantly, a novel PLAG1::FTO gene fusion was identified in this case. Our findings not only highlight the diagnostic complexity of this rare breast neoplasm, which is prone to misclassification, but also expand the known genetic landscape of PA through the discovery of the novel PLAG1::FTO fusion. This study thus provides valuable new insights for future molecular research on pleomorphic adenoma.

## Introduction

Pleomorphic adenoma (PA), is the most commonly encountered benign neoplasm observed in the salivary glands. In contrast, Its manifestation in the breast is extremely rare, with only isolated case reports and small series having been documented (1–8). It may easily be overlooked in breast pathology, resulting in missed diagnosis, particularly when evaluating frozen sections or needle biopsy samples. The abundant myxoid stroma can mimic mucinous carcinoma or matrix-producing variants of metaplastic carcinoma, particularly on limited sampling (5, 9). As a result, there is a risk of misdiagnosis and potential overtreatment. *PLAG1 or HMGA2* fusion is currently the most widely recognized genetic event in salivary gland PA, but molecular research on breast PA remains relatively rudimentary, with published studies having documented the same morphologic features as in corresponding salivary neoplasms. Here, we report for the first time a case of breast PA with *PLAG1::FTO* fusion and describe its clinical, pathological, and molecular features.

## Materials and methods

### Clinical presentation

**Figure 1:**
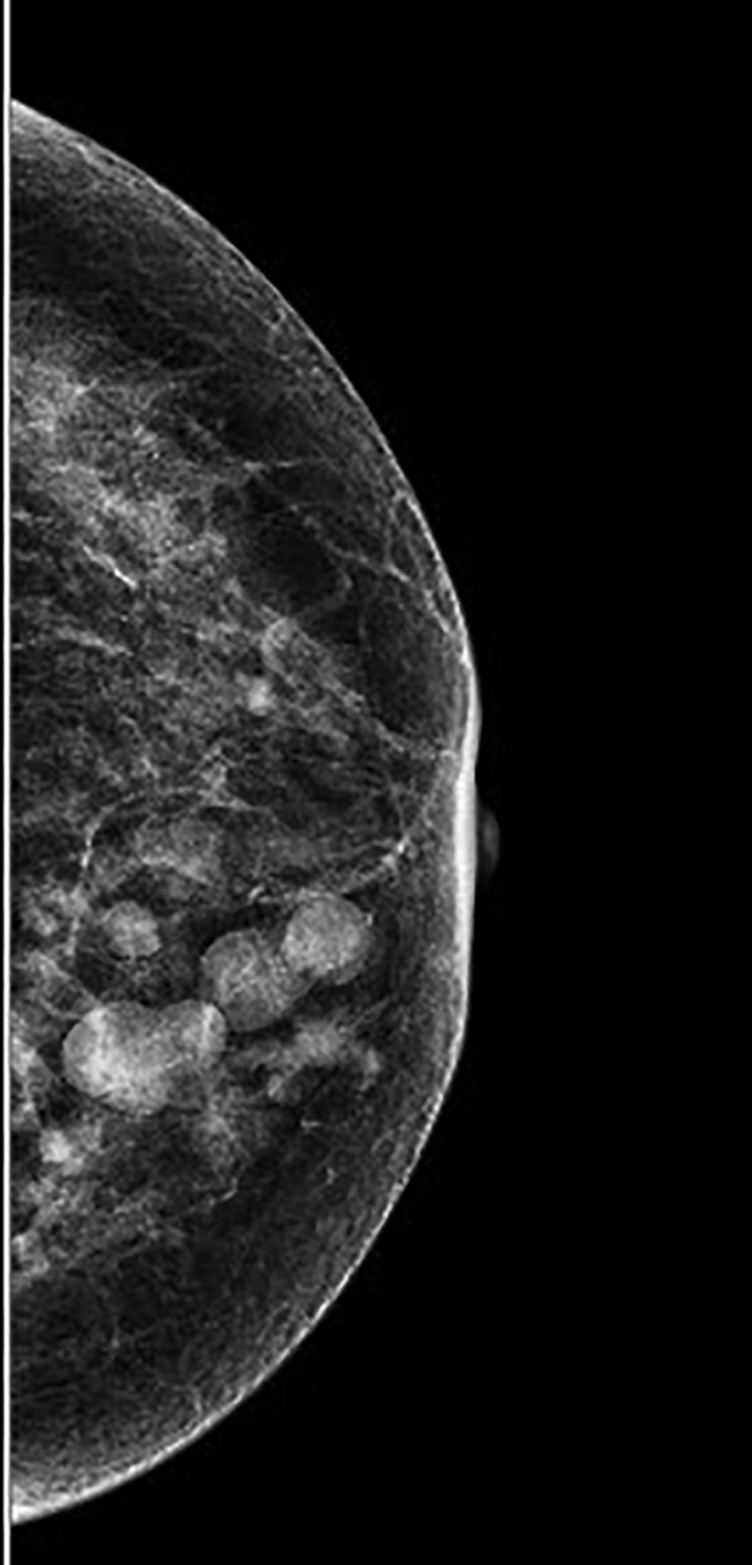
Mammography: Multiple small and large round nodules of different sizes were found in the lower inner quadrant of the left breast, distributed in a segmental pattern along the ducts.

A 59-year-old woman presented with palpated nodules in the left breast, which she had identified on self-examination. Physical examination revealed several non-tender nodules in the lower inner quadrant of the left breast without overlying skin changes, nipple discharge, or ulceration. Mammographic imaging revealed multiple rounded opacities with segmental distribution along the ductal system, the largest measuring 1.9 × 1.3 cm (**Figure 1**). Targeted breast ultrasound demonstrated multiple well-circumscribed, complex cystic-solid nodules with heterogeneous internal echotexture, localized to the 6–7 o’clock, 8–9 o’clock, and 9 o’clock periareolar regions. The largest measured 1.07 × 0.902 cm, with others ranging between 0.336 cm and 0.765 cm in maximal dimension.

### Immunohistochemistry

Representative formalin-fixed, paraffin-embedded tissue samples were selected, and 4 μm-thick sections were stained with antibodies specific for estrogen receptor (ER, clone: SP1, Abcam), progesterone receptor (PR, clone: SP2, Abcam), CK5/6 (clone: D5/16B4, DAKO), HER2 (clone: 4B5, Roche); p63 (clone: 4A4, Gene), S100 (clone: 4C4.9, Abcam), Calponin (clone: EP798Y, LBPMedicin), and Ki67 (clone: MIB-1, DAKO).

### Hi-C library preparation

Hi-C sample preparation and library preparation were performed by Abcepta. Briefly, 3–5 FFPE tissue sections (10 μm thick) were deparaffinized and incubated in 0.3% SDS at 65 °C for 30 minutes, followed by quenching with 3% Triton X-100 at 37 °C for 15 minutes. The samples were then washed and resuspended in digestion buffer. Chromatin was digested with 200 U of DpnII restriction enzyme at 37 °C overnight with gentle rotation. Subsequently, dCTP, dGTP, dTTP, biotin-14-dATP, and Klenow DNA polymerase (5 U/μL) were added, followed by incubation at 23 °C for 4 h with rotation. The digested chromatin was ligated with 400 U T4 DNA ligase at 16 °C overnight. DNA was then isolated, and unligated ends were treated with T4 DNA polymerase to remove biotin labeling.

Up to 1 μg of DNA was fragmented, followed by end repair, A-tailing, and purification. The ligation products were enriched using 10 μl streptavidin beads and used as input for library preparation. Final libraries were quantified with a Qubit 4.0 Fluorometer (Thermo Fisher) and Qsep100 (BiOptic Inc.), followed by sequencing on an MGI DNBSEQ-T7 platform (30–50 million paired-end reads/sample).

### RNA sequencing

Total RNA from each sample was extracted with an RNA extraction kit used as directed. RNA quality and concentration were evaluated with Qubit 4.0 Fluorometer and Nanodrop One (Thermo Fisher Scientific) instruments. Libraries were prepared following the standard protocol (catalog number, Vazyme), which included cDNA synthesis, A-tailing, adapter ligation, and PCR amplification. Probes were designed for a panel of 180 genes often fused in common solid tumors to capture the targeted regions of interest with the probeCap Hybridization and Wash Kit (catalog number, Homgen). Pair-end sequencing was performed using an MGI DNBSEQ-T7 platform, yielding 15–30 million reads.

## Results

### Frozen section

**Figure 2:**
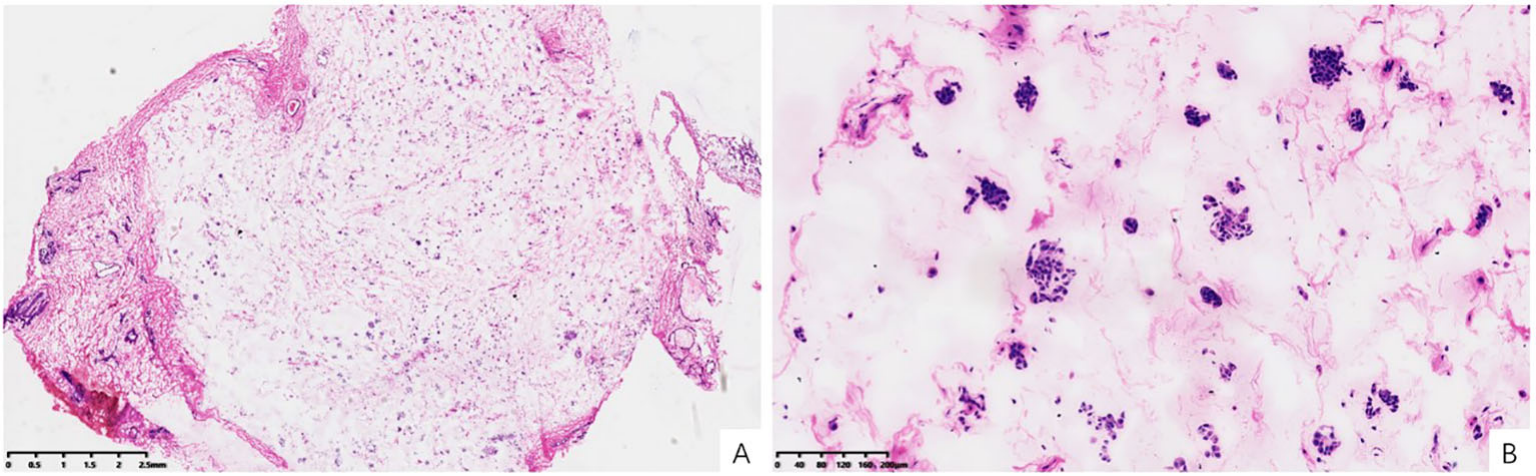
Frozen section. **(A)** Neoplasm with prominent mucin secretion (H&E, x10). **(B)** Small clustered or nest-like cells floating within the myxoid stroma (H&E, x100).

The patient underwent lumpectomy of the left breast. The gross specimen revealed multiple well-circumscribed nodules ranging from 0.3 cm to 1.9 cm in greatest dimension, with a firm, gray-white cut surface and glistening myxoid areas. Sections from representative nodules revealed a mucin-rich neoplasm composed of small nests and clusters of uniform epithelial cells suspended in a myxoid background (**Figure 2A**). No significant cytological atypia, mitotic figures, or necrosis were observed (**Figure 2B**). Nevertheless, due to the prominent mucin and architectural pattern, the preliminary diagnosis suggested a “mucin-producing tumor” favoring a mucinous carcinoma.

### Paraffin section

**Figure 3:**
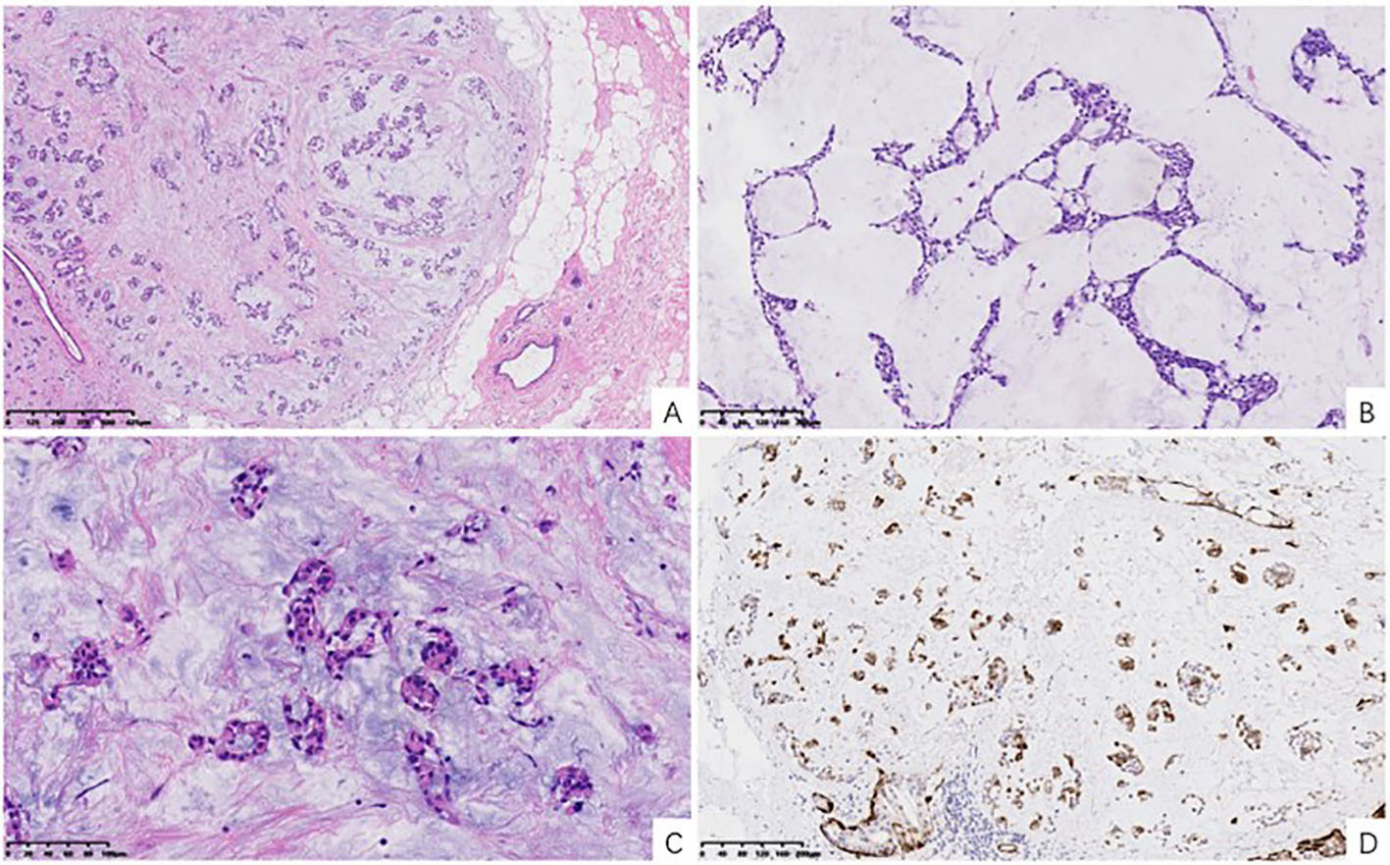
Histopathological features of mammary pleomorphic adenoma. **(A)** Low-power magnification showing a well-circumscribed, multinodular tumor with a fibrous pseudocapsule (H&E, x40). **(B)** Medium-power magnification demonstrating diverse architectural features, including tubular structures and solid nests within a myxoid background (H&E, x100). **(C)** High-power magnification highlighting the characteristic chondromyxoid stroma with chondrocyte-like cells (H&E, x200). **(D)** Immunohistochemistry staining for Calponin, highlighting the prominent myoepithelial cell component surrounding epithelial nests and dispersed within the stroma (Calponin IHC, x100).

Histologic evaluation of paraffin-embedded sections demonstrated a multinodular, well-demarcated lesion separated from adjacent breast tissue by variably thick fibrous pseudocapsules (**Figure 3A**). The neoplasm exhibited a range of architectural configurations, including tubules, cribriform islands, solid nests, and cords, intermixed within a strikingly myxoid to chondromyxoid stroma (**Figures 3B, C**). The epithelial component featured bland cuboidal cells with uniform nuclei, while the myoepithelial component appeared as spindled or stellate cells dispersed within the stroma or enveloping epithelial structures. There was no evidence of mitotic activity, necrosis, perineural invasion, or lymphovascular infiltration.

### Immunohistochemistry

Immunostaining confirmed the biphasic cellular nature of the tumor. Cytokeratin staining highlighted epithelial tubular and ductal structures. P63 and Calponin strongly labeled the myoepithelial cells both around glandular units and within the stroma (**Figure 3D**), with diffuse positivity. S100 protein staining revealed diffuse nuclear and cytoplasmic staining in stromal and chondroid areas. Estrogen receptor (ER), progesterone receptor (PR), and HER2 staining results were all negative. The Ki-67 proliferation index was approximately 5%.

### Molecular analysis

**Figure 4:**
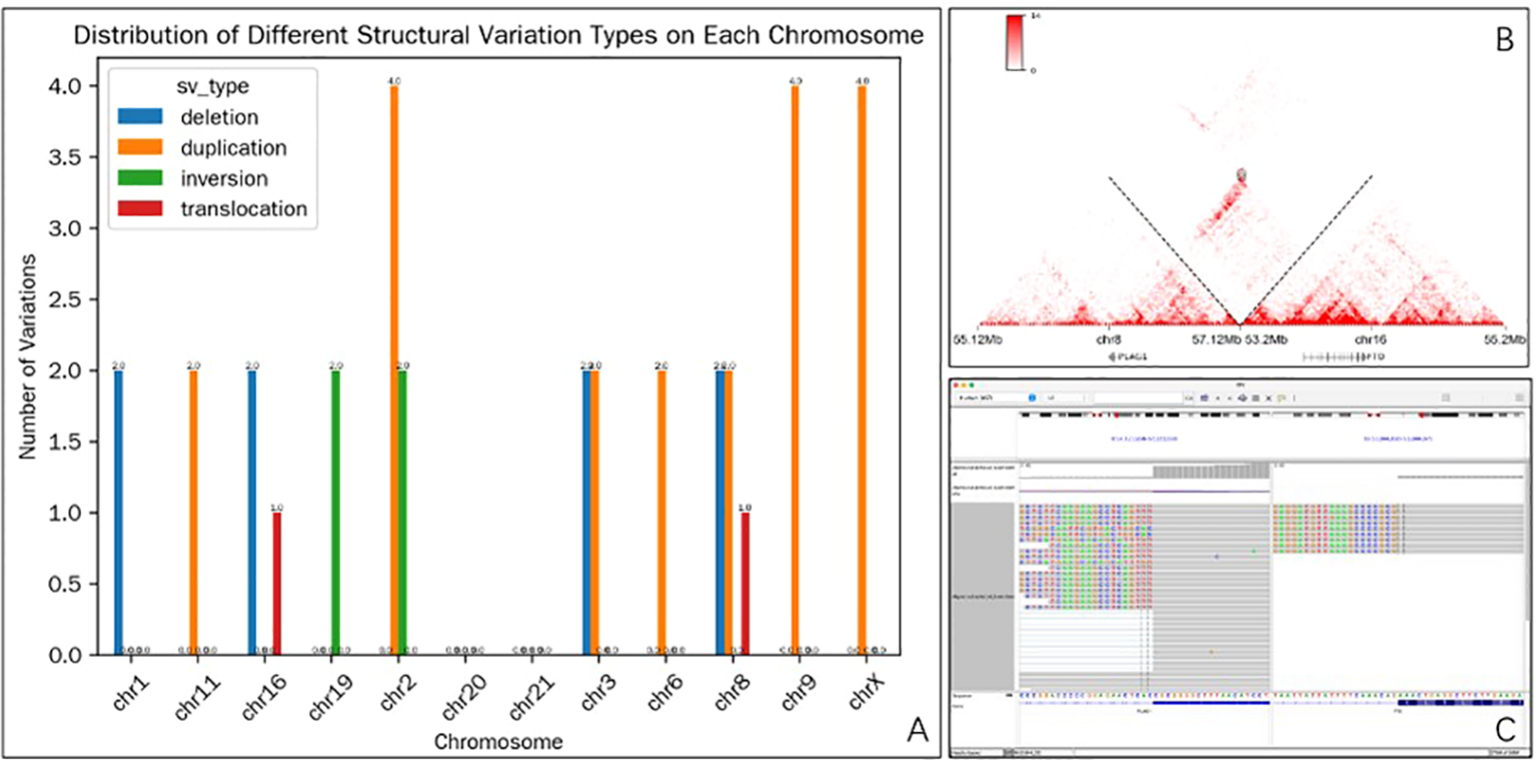
Characterization of the *PLAG1::FTO* translocation. **(A)** Distribution of Different Structural variant types on each chromosome. **(B)** Hi-C analysis (10-kb resolution) identifying a robust balanced translocation event between the *PLAG1* locus (chromosome 8) and the *FTO* locus (chromosome 16). **(C)** IGV visualization depicting the transcriptional read alignment patterns from RNA sequencing data, supporting the translocation event.

Hi-C was employed to identify the distribution of different structural variation types on each chromosome (**Figure 4A**), revealing the presence of a novel *PLAG1::FTO* gene translocation variant on chromosomes 16 and 8 (**Figure 4B**). Cross-validation via RNA-seq confirmed the fusion event (**Figure 4C**).

### Treatment and outcome

Following lumpectomy, the case was successfully followed up for ten months, with no recurrence or metastasis.

## Discussion

PA of the breast, initially described by Smith and Taylor in 1969 (1), is an exceedingly uncommon clinical entity. It predominantly affects postmenopausal women, with a reported female-to-male ratio of approximately 10:1, although isolated cases in males have been documented (3, 8). The pathogenesis of mammary PA is not fully elucidated, but some authors propose that it may arise from intraductal papillomas through exaggerated myoepithelial proliferation and stromal matrix production. Multifocal occurrences, as observed in this case, have also been linked to the presence of multiple underlying papillomatous foci (8, 10, 11).

Consistent with its “pleomorphic” designation, the histological heterogeneity of PA can present significant diagnostic challenges, particularly when working with frozen sections, fine needle aspirates, or core biopsy specimens (5, 9, 12). The present case illustrates the diagnostic complexities associated with mammary PA. Microscopically, the lesion consisted of well-formed glands and cords lined by an inner layer of epithelial cells and an outer layer of myoepithelial cells, set within an abundant myxoid matrix. Notably, the tumor lacked significant cytologic atypia, mitotic activity, or necrosis. While mucinous carcinoma typically exhibits cohesive clusters of bland epithelial cells floating in lakes of mucin with minimal stromal architecture. Immunohistochemistry played a vital role in diagnosing this case, highlighting myoepithelial elements with Calponin and P63, and confirming stromal myoepithelial presence via S100 positivity. The absence of ER, PR, and HER2 expression, along with a low Ki-67 proliferation index, further supported the benign nature of this neoplasm. A uniformly myxoid stroma may falsely suggest mucinous carcinoma, but the presence of stromal myoepithelial cells and biphasic architecture are not typical of mucinous carcinoma.

Other key entities to consider in the differential diagnosis include matrix-producing metaplastic carcinoma, adenomyoepithelioma, papilloma with cartilaginous metaplasia, and myxoid fibroadenoma. Importantly, breast carcinosarcoma or metaplastic carcinoma may mimic PA histologically due to the presence of heterologous elements and myxoid stroma; however, these lesions are malignant and typically demonstrate significant cytologic atypia, high mitotic activity, and different molecular profiles, such as TP53 mutations, PI3K pathway mutations (PIK3CA, PTEN loss), and complex karyotypes, rather than the specific PLAG1 or HMGA2 rearrangements characteristic of PA. Similarly, benign phyllodes tumor can show stromal overgrowth and heterologous cartilaginous or osseous elements, but it is genetically distinct, typically harboring MED12 mutations, TERT promoter mutations, or RARA rearrangements instead of PLAG1/HMGA2 fusions. In this case, the absence of nuclear atypia and low mitotic activity helped exclude matrix-producing metaplastic carcinoma. Although adenomyoepithelioma also exhibits dual epithelial-myoepithelial differentiation, it typically features a prominent and often stratified myoepithelial layer with less chondroid stroma. Salivary gland-type carcinomas such as adenoid cystic carcinoma (ACC) and mucoepidermoid carcinoma (MEC) may enter the differential. Neither exhibits the hallmark chondromyxoid stroma or PLAG1 gene rearrangement characteristic of PA. These molecular differences are summarized in **Table 1**, which highlights the distinct genetic landscapes of salivary gland-like tumors and their differential diagnoses.

**Table 1:** Recurrent molecular genetic alterations in salivary gland-like tumors of the breast.

Tumor type	Recurrent molecular alterations	Key features
Pleomorphic adenoma	PLAG1 rearrangements (PLAG1::FTO, PLAG1::CTNNB1, PLAG1::TRPS1, etc.), HMGA2 rearrangements (HMGA2::WIF1)	Benign; biphasic differentiation; chondromyxoid stroma
Mucoepidermoid carcinoma	CRTC1::MAML2, CRTC3::MAML2 fusions	Malignant; epidermoid/intermediate cells; mucous cells
Adenoid cystic carcinoma	MYB::NFIB, MYBL1 rearrangements	Malignant; cribriform/tubular pattern; basaloid cells
Adenomyoepithelioma	HMGA2::WIF1 (subset), PLAG1 rearrangements (subset)	Dual differentiation; prominent myoepithelial layer
Secretory carcinoma	ETV6::NTRK3 fusion	Malignant; S100+, mammary-type secretion
Carcinosarcoma/Metaplastic carcinoma	TP53 mutations, PIK3CA mutations, PTEN loss, complex karyotypes	Malignant; heterologous elements; high-grade atypia
Benign phyllodes tumor	MED12 mutations, TERT promoter mutations, RARA rearrangements	Leaf-like architecture; stromal overgrowth; no PLAG1/HMGA2

Genetically, salivary gland PA frequently harbor chromosomal rearrangements involving PLAG1 or HMGA2. Recent studies have demonstrated that breast PAs share similar genetic alterations with their salivary gland counterparts, including fusions such as HMGA2::WIF1, CTNNB1::PLAG1, and TRPS1::PLAG1 in mammary PA (6, 7). However, the full spectrum of molecular alterations in breast PA remains to be fully characterized, and further studies are needed to determine whether all genetic changes defining salivary gland PA are also present in breast PA. Interestingly, *HMGA2::WIF1* has also been detected in a subset of adenomyoepitheliomas, suggesting potential overlap in pathogenesis or derivation from a common progenitor lesion (13). In the present report, Hi-C was used to identify the distribution of different structural variants across chromosomes (including chromosomes 1, 11, 16, 19, 2, 3, 6, 7, 9, and X). Different chromosomes exhibited distinct frequencies of each structural variant type. For instance, chr2 presented with relatively high numbers of duplication (4 occurrences) and inversion (2 occurrences) events, while translocation was relatively rare and only appeared on chr16 and chr8. Cross-validation via RNA-seq confirmed the identified fusion event. *PLAG1* encodes a zinc finger protein associated with cell cycle progression that can become activated as a result of promoter exchange with a variety of different partner fusion genes. When overexpressed, *PLAG1* target genes tend to become dysregulated, most importantly *IGF2*, thereby driving tumorigenesis through transcriptional activation and altered MAPK pathway signaling. The novel *PLAG1::FTO* fusion gene is likely associated with the development of this breast PA lesion, further expanding the molecular spectrum associated with this rare breast tumor type. While the presence of this fusion was confirmed by both Hi-C and RNA-seq, the inability to perform PLAG1 immunohistochemistry limits our ability to demonstrate PLAG1 protein overexpression at the translational level. Therefore, the pathogenic significance of this specific fusion requires validation in additional cases and functional studies, which we acknowledge as a limitation of the current study.

Surgical excision with negative margins remains the standard treatment for breast PA (14–16). The prognosis is generally favorable, though incomplete excision may lead to local recurrence. Rare instances of malignant transformation have been reported, prompting some investigators to classify PA as a neoplasm of uncertain malignant potential (5). In light of this, we recommend long-term surveillance and consider molecular testing in diagnostically uncertain or recurrent cases. Further research is needed to define the clinical and prognostic significance of specific molecular alterations, such as the *PLAG1::FTO* fusion described here.

## Data Availability

The raw data supporting the conclusions of this article will be made available by the authors, without undue reservation.
